# Annexin-Mediated Calcium Signalling in Plants

**DOI:** 10.3390/plants3010128

**Published:** 2014-02-26

**Authors:** Julia M. Davies

**Affiliations:** Department of Plant Sciences, University of Cambridge, Cambridge CB2 3EA, UK; E-Mail: jmd32@cam.ac.uk; Tel.: +44-1223-333-939; Fax: +44-1223-333-953

**Keywords:** annexin, *Arabidopsis*, calcium, channel, *Medicago*, signal, stress

## Abstract

Calcium-permeable channels underpin elevations of free calcium that encode specific signals in stress adaptation, development and immunity. Identifying the genes encoding these channels remains a central goal of plant signalling research. Evidence now suggests that members of the plant annexin family function as unconventional calcium-permeable channels, with roles in development and stress signalling. Arabidopsis annexin 1 mediates a plasma membrane calcium-permeable conductance in roots that is activated by reactive oxygen species. Recombinant annexin 1 forms a very similar conductance in planar lipid bilayers, indicating that this protein could facilitate the *in vivo* conductance directly. The annexin 1 mutant is impaired in salinity-induced calcium signalling. Protein–protein interactions, post-translational modification and dynamic association with membranes could all influence annexin-mediated calcium signalling and are reviewed here. The prospect of annexins playing roles in calcium signalling events in symbiosis and immunity are considered.

## 1. Introduction

Calcium influx to the cytosol (from the apoplast or from organellar stores) is central to elevation of cytosolic free Ca^2+^ ([Ca^2+^]_cyt_) as a second messenger in developmental, stress and immune signalling [1]. Elevation of free Ca^2+^ within some organelles could also have a signalling role. Gradually, the Ca^2+^-permeable channels involved in the stimulus-specific, transient free Ca^2+^ elevations or oscillations are being identified (reviewed by [2,3]). At the vacuole of *Arabidopsis thaliana* and rice, TPC1 (Two Pore Channel 1) would be capable of releasing Ca^2+^ to the cytosol [4], although modelling studies suggest that it would not be a component of guard cell Ca^2+^ signalling for aperture control [5]. At the plasma membrane, members of the Glutamate Receptor-Like (GLR) family of ion channel sub-units mediate Ca^2+^ influx into pollen tubes and root cells [6,7]. In pollen tubes, they co-reside with members of the Cyclic Nucleotide-Gated Channel (CNGC) family [8]. AtCNGC2 is the best studied of the family and lies downstream of specific receptors for defence responses [9,10]. All of these channels are “conventional” in that each gene encodes an integral, trans-membrane spanning subunit with a pore-forming loop that is targeted to a specific membrane and is most likely united with other subunits to form a functional channel.

Recent studies have shown that some Ca^2+^ influx pathways may not be formed by conventional channels. There is scope for passive Ca^2+^ transport mediated by annexins [11,12]. These small amphipathic proteins are distributed throughout cells (reviewed by [13]) and can be transported within the plant via the phloem [14]. Expression can be regulated by Ca^2+^ [15]. There is now strong evidence for plant annexins’ forming Ca^2+^-permeable transport pathways across bilayers *in vitro*. Results from an annexin loss of function mutant are consistent with an *in vivo* Ca^2+^ transport function and further studies are now needed to establish how annexins could directly mediate Ca^2+^ transport in native membranes. It is possible that annexins could be recruited directly to membranes, independently of vesicle delivery, to operate in stimulus-specific signalling. This short review will introduce this family of Ca^2+^-binding proteins and address what is known about their role in Ca^2+^ signalling in plants.

## 2. Ubiquitous Annexins

Genome studies have revealed that higher plants harbour multi-gene annexin families; eight in *Arabidopsis*, ten in rice, and twenty-three in soybean [16,17]. Excellent reviews by Clark *et al*. [16] and Jami *et al*. [18] address their phylogeny and evolution. These small (32 to 42 kDa) proteins are expressed throughout the higher plant body, with expression varying with development and environmental conditions, including light, water availability, temperature, salinity, acid rain, gravity, metal stress, mechanical stress, presence of microbes and nutrient deprivation [13,19,20,21,22,23,24,25,26]. Transcriptional regulators are now being identified, such as MYB98 and UPB1 in *Arabidopsis thaliana* [27,28]. Analyses of expression and protein abundance have revealed widespread distribution of annexins through the plant, with greater abundance at growth points such as root hair apices (reviewed by [13,16]). Distribution through the plant via the phloem also appears likely [14]. Unlike conventional transport proteins, an annexin can ostensibly exist in the cytosol or extracellular matrix, in addition to being membrane associated or inserted. The clearest example is *Arabidopsis* annexin 1 (AtANN1; At1g35720). In addition to a predominant presence in the cytosol, proteomic, immunolocalisation, radiolabelling and GFP studies have identified AtANN1 at the plasma membrane (as an integral protein), ER, vacuole, mitochondria, chloroplast, in phloem exudate and cell wall [14,29,30]. How an annexin becomes extracellular is unknown but the AtANN1 sequence is consistent with its being a non-classical secreted protein [31] and it also harbours a diacidic motif that should target it to the plasma membrane [13]. By analogy with animal annexins, export could also be via exocytosis or ABC transporters (reviewed by [13]). Extracellular animal annexin function can be through receptor binding [32] but this remains to be explored for plant annexins.

The mechanistic basis of membrane association is far better understood but still lags behind that of animal annexins. Plant annexins contain up to four “annexin repeats” that would facilitate reversible Ca^2+^-dependent binding to negatively charged phospholipid head groups [33,34]. Half maximal binding requires nanomolar to millimolar Ca^2+^ (reviewed by [13]). Membrane binding may also involve the *N*-terminus. Ca^2+^-independent binding to lipids is possible at neutral and acidic pH [31,35,36]. In common with animal annexins, plant annexins can fully or partially insert into membranes, with clear examples coming from wheat and *Arabidopsis* [35,37]. Some animal annexins support Ca^2+^ channel-like behaviour *in vitro* by inserting into or associating with the bilayer, with transport activity regulated by ATP, GTP, peroxide, pH and voltage (reviewed by [13,38]. Given the structural similarity of plant annexins to their animal counterparts, including the conservation of salt bridges implicated in channel selectivity and regulation [13,31,38], it is reasonable to hypothesise that plant annexins capable of membrane association or insertion could act as Ca^2+^-permeable channels *in vivo* and so have a role in Ca^2+^ signalling.

## 3. Ca^2+^ Transport by Plant Annexins

The first indication that plant annexins could form Ca^2+^-permeable transport pathways came from the incorporation of (recombinant) *Capsicum annuum* CaANN24 into vesicles containing a Ca^2+^ indicator dye [33]. Since then, purified native *Zea mays* annexins ZmANN33/35 were found to increase [Ca^2+^]_cyt_ when added to the extracellular membrane face of *Arabidopsis* root protoplasts as a bioassay [31]. This indicated that extracellular annexins could somehow modulate [Ca^2+^]_cyt_ but whether this was by directly forming a Ca^2+^ influx pathway or through activation of other channels was not determined [31]. More tellingly, ZmANN33/35 formed a Ca^2+^- and K^+^-permeable conductance when added to the equivalent of the cytosolic face of a planar lipid bilayer, designed to act as a plasma membrane mimetic [31]. This conductance was blocked by the cation channel blocker Gd^3+^ present at the equivalent extracellular face of the bilayer, indicating that a trans-bilayer conductance had been formed by the annexins [31]. Incubating the annexins with their cognate antibody prevented the formation of the conductance. The incorporation of malondialdehyde (MDA) into the planar lipid bilayer to mimic lipid peroxidation caused a profound change in the way the ZmANN33/35 conductance was regulated by voltage. MDA forms in membranes during stress responses known to involve reactive oxygen species (ROS) and [Ca^2+^]_cyt_ elevation [39]. In control conditions the annexin-mediated conductance increased in a linear fashion as voltage became more negative (hyperpolarised). In contrast, MDA restricted annexin Ca^2+^ transport activity to more hyperpolarised voltages and this could in turn have implications for a resultant [Ca^2+^]_cyt_ signal *in vivo* [31,39]. The mechanism for this change in voltage sensitivity is also unknown but the channel-forming animal annexin A5 can bind to MDA and is implicated in Ca^2+^ influx across the plasma membrane in response to hydrogen peroxide [40,41]. Perhaps binding of one or both of the *Zea* annexins to MDA effected voltage regulation.

Work on ZmANN33/35 in bilayers established that the conductance was selective for K^+^ over Ca^2+^ [31]. Single channel behaviour (discrete, step changes in current translocated across the bilayer) has rarely been observed but a single channel conductance of 17 pS was recorded in MDA-containing bilayers [38]. Recombinant *Medicago truncatula* Annexin 1 (MtANN1) has recently been shown to support single channel activity in planar lipid bilayers [42]. When transporting K^+^, three different single channel conductances were observed; 16pS, 135 pS and 329 pS depending on the amount of annexin present. This is similar to channel behaviour shown by animal annexins in bilayers, where amount of protein is linked to level of oligomerization, association with the bilayer and channel characteristics such as conductance and voltage dependence [13,29,38,42,43]. Testing for Ca^2+^ transport by MtANN1 is now feasible.

Further progress has been made on the transport activity and function of the predominantly abundant annexin of Arabidopsis, AtANN1. Recombinant AtANN1 was first reported to form a K^+^ conductance in planar lipid bilayers by Gorecka *et al*. [36], with activity promoted by acidic pH. Exposure to Ca^2+^ was found to prevent transport activity unless the planar lipid bilayer was itself exposed to copper and ascorbate to generate hydroxyl radicals [11]. In these experiments, AtANN1 was present at the cytosolic face of a plasma membrane mimetic bilayer and hydroxyl radicals (OH^●^) were generated at the extracellular face. OH^●^ are the most potent and short-lived of the ROS. Why Ca^2+^ was inhibitory to AtANN1 transport function and how OH^●^ overcame this now need to be elucidated. In common with ZmANN33/35, Gd^3+^ at the “extracellular” bilayer face blocked the AtANN1-mediated conductance thus indicating that a trans-bilayer transport pathway had been formed and conductance formation was prevented by incubation with anti-AtANN1 antibody [11]. Analysis of ionic selectivity has revealed that although only modestly permeable to Ca^2+^, the OH^●^-activated AtANN1 conductance discriminates between K^+^ and Na^+^ very well; the Ca^2+^:K^+^ selectivity ratio is 0.64, Ca^2+^:Na^+^ is 11 and the K^+^:Na^+^ is 18 [11,12].

Plant annexins have been found to have *in vitro* ATPase and GTPase activity (reviewed by [13,44]. AtANN1 has been identified *in vitro* as an ATP-binding protein [45]. This has led to the proposal that it may be involved in [Ca^2+^]_cyt_ elevations caused by extracellular ATP [13,46]. Both extracellular ATP and ADP cause transient elevation of [Ca^2+^]_cyt_ in plant cells and help regulate growth, stress and immune responses [47,48,49]. However, plant genomes do not contain the equivalent genes encoding animal ATP/ADP receptors [49]. The capacity of AtANN1 to be extracellular, bind ATP and form a Ca^2+^ transport route makes it a candidate for the plant’s functional equivalent of those receptors [13,46]. Although there are still no reports on AtANN1, recently the Na^+^ transport activity of recombinant MtANN1 in planar lipid bilayers was reported to be promoted by ATP (data not shown in [42]).

While bilayer studies have clearly shown the capacity of annexins to translocate Ca^2+^ and K^+^, few studies have addressed *in vivo* transport function. As, in common with animal annexins, plant annexins are firmly implicated in exocytosis [50], analysis of loss of function mutants may not yield clear-cut results. The absence of a conductance could be due to a failure in annexin-mediated exocytotic delivery of a channel subunit to a membrane rather than a failure in annexin-mediated ion transport itself. In fairness, there are caveats also to the interpretation of conventional channel mutants; for example, loss of transport function could be due to a pleiotropic effect of the mutation. The safeguard is to examine the protein’s transport activity *in vitro*, without the confounding effects of a cellular expression system, whether native or heterologous. Does the *in vitro* transport match that of the membrane?

## 4. *In Vivo* Activity and Functions of AtANN1

The root epidermal and root hair apical plasma membrane of *Arabidopsis* contain a hyperpolarisation-activated Ca^2+^-permeable channel conductance that is activated by extracellular hydroxyl radicals (OH^●^) and is involved in growth [11,51,52]. Using patch clamp electrophysiology, an *Atann1* knockout mutant was found to lack this Ca^2+^ conductance in both epidermal and root hair apical plasma membrane, with activity restored by complementation. The transport characteristics of the OH^●^-activated Ca^2+^ conductance generated by recombinant AtANN1 in planar lipid bilayers agree well with that of the native membrane, strongly supporting AtANN1’s direct formation of this transport pathway. Root epidermal protoplasts from the mutant were significantly impaired in their ability to elevate [Ca^2+^]_cyt_ in response to extracellular OH^●^, consistent with a Ca^2+^ transport function for AtANN1 [11]. Mutant roots and root hairs were significantly shorter than wild type, consistent with impaired Ca^2+^ uptake [11].

Recent work has shown that AtANN1 is involved in root [Ca^2+^]_cyt_ elevation in response to hydrogen peroxide, using aequorin as a [Ca^2+^]_cyt_ reporter [53]. H_2_O_2_ evokes a markedly different [Ca^2+^]_cyt_ response to extracellular OH^●^ both in whole roots and root epidermal protoplasts, indicating that different ROS can generate specific [Ca^2+^]_cyt_ signals. In addition to having a role in H_2_O_2_-induced Ca^2+^ influx, AtANN1 was implicated in mediating Ca^2+^ release from intracellular stores when plasma membrane influx was blocked by Gd^3+^. Although this may not be physiologically relevant, it helps make some sense of AtANN1’s reported associations with endomembranes and shows that it can respond to perturbation of [Ca^2+^]_cyt_ homeostasis. With no block of influx by Gd^3+^, loss of AtANN1 function perturbed and diminished the H_2_O_2_-induced [Ca^2+^]_cyt_ signal leading to impaired transcription of *Glutathione-S-Transferase1 Tau 1* (*GST1*; [53]). This upregulation of *AtGST1* by H_2_O_2_ was shown previously to be dependent on Ca^2+^ influx [54]. The mode of AtANN1’s action in this system is unknown but H_2_O_2_ causes AtANN1 to dimerise *in vitro* [55].

**Figure 1:**
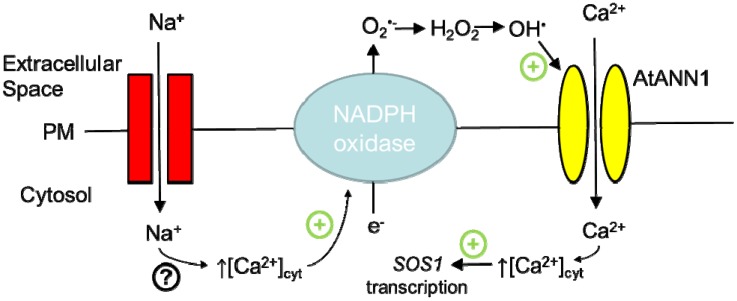
Functional NADPH oxidase/annexin unit in calcium signalling. Studies with *Arabidopsis* suggest that annexin 1 (AtANN1) can function downstream of a plasma membrane NADPH oxidase in salinity stress. In roots, Na^+^ entry across the plasma membrane (PM) causes elevation of cytosolic free Ca^2+^ which could activate NADPH oxidase through the latter’s EF hands (helix-loop-helix structural domains usually involved in Ca^2+^ binding). This would result in extracellular ROS production that would activate AtANN1-mediated Ca^2+^ influx to promote SOS1 transcription and secondary root formation [11,12,58].

As an OH^●^-activated plasma membrane Ca^2+^ conductance, AtANN1 is expected to operate downstream of plasma membrane NADPH oxidases, the activity of which can ultimately source extracellular OH^●^ [52,56]. Specifically, AtANN1 is likely to operate with the NADPH oxidase encoded by AtRBOHC (Respiratory Burst Oxidase Homologue C), which operates in root growth and salinity stress signalling [52,57]. The ROS sourced by AtRBOHC stabilise transcript for the plasma membrane Na^+^/H^+^ antiporter, SOS1 that is fundamental to resisting salinity stress and this stabilisation also requires Ca^2+^ influx [57]. The *Atann1* loss of function mutant fails to activate the root epidermal plasma membrane Ca^2+^ influx conductance in response to salinity stress (Figure 1) [12].

Moreover, AtANN1 underpins the salinity induced [Ca^2+^]_cyt_ elevation in root epidermal protoplasts that requires oxidation, consistent with its acting as an ROS-activated Ca^2+^ influx conductance [12]. In this respect, the low Na^+^ permeability of recombinant AtANN1 in planar lipid bilayers makes biological sense because it could act downstream of AtRBOHC to amplify the [Ca^2+^]_cyt_ signal without exposing the root to further Na^+^ influx. There is a profound NaCl-induced recruitment of AtANN1 to membranes [57] and while some of this may be for Ca^2+^ signalling, it may be an attempt by the plant to inhibit Na^+^ ingress and K^+^ loss. Loss of AtANN1 function results in significantly increased Na^+^ influx and K^+^ efflux from roots [12]. How AtANN1 functions as a negative regulator of these transport reactions and the identities of these important transport proteins now need to be determined. Downstream of the impaired [Ca^2+^]_cyt_ signal in the *Atann1* mutant was a failure to increase *AtSOS1* transcription and a significant reduction in the production of secondary roots in response to salinity stress [12].

## 5. Regulation of Annexin Positioning

Positioning within the cell or extracellularly will most certainly prove to be of fundamental importance to annexin function in Ca^2+^ signalling, whether acting as a transport pathway or not. For animal annexins, contact with membranes is regulated by various factors including pH, voltage, [Ca^2+^]_cyt_, membrane curvature and specificity of binding to phospholipid headgroups (reviewed by [13]). Cold, salinity and mechanical stress cause repositioning of plant annexins, often to membranes [35,57,59,60] while infection by *Phytophthora* causes secretion of a potato annexin [21].

Recently, AtANN1 has been detected in a detergent resistant plasma membrane “nanodomain” involved in mesophyll ABA signalling [61]. Its role there is unknown and while it could directly contribute to ABA-induced [Ca^2+^]_cyt_ signalling as a transporter, work on animal annexins has shown involvement in formation of such domains [62]. MtANN2 has also been recovered from a detergent resistant plasma membrane domain, where it resided with an NADPH oxidase [63]. Detergent-resistant domains hold NADPH oxidases at the apex of pollen tubes [64] and this may also be the case for root hairs. The co-localisation of NADPH oxidases with annexins as ROS-activated Ca^2+^ transporters would afford tight spatial specificity of [Ca^2+^]_cyt_ elevation in polar growth of pollen tubes and root hairs. Lipid composition of the extracellular face of the plasma membrane could recruit extracellular annexins to act in [Ca^2+^]_cyt_ signalling. During plant cell death, phosphatidylserine everts to the extracellular leaflet of the plasma membrane [65,66]. The ability of extracellular ZmANN33/35 to elevate [Ca^2+^]_cyt_ has been hypothesised to be involved in cell death, which would be consistent with the ability of annexins to bind phosphatidylserine [31].

## 6. Interacting Proteins

Interaction with other proteins has been reviewed by [13] but the bearing of such interactions on Ca^2+^-mediated signalling remains to be determined. Annexin-annexin interaction could influence a signalling function. Peroxide as a key component of ROS signalling, causes *in vitro* oligomerisation of ZmANN33/35 [44] and dimerisation of AtANN1 [55]. AtANN1 can interact with AtANN4, possibly to function in drought stress, but the impact of this on AtANN1’s role in [Ca^2+^]_cyt_ signalling remains to be tested [67]. Recently, sea cotton (*Gossypium barbadense*) annexins 5 and 6 have been shown to form homodimers and heterodimers [68]. GbANN6 was found at the plasma membrane and nucleolus when heterologously expressed and was also found to bind F-actin. Tomato and mimosa annexins also bind actin while annexin expression is now clearly involved in cotton fibre elongation [68,69]. Expression of *GbANN6* in *Arabidopsis* increased root cell length, which was positively correlated with amount and position of F-actin [68]. These results have led to the proposal of GbANN6’s acting as a scaffolding protein [68]. It will be interesting to see whether annexin-actin interaction has any bearing on actin regulation of the plasma membrane hyperpolarisation-activated Ca^2+^ channels involved in pollen viability and growth [69,70].

Interaction with C2 domain-containing proteins via an K/H/RGD motif has been proposed, which would implicate annexins in regulation of such signalling components as phospholipases [13,16,18,71]. *In vitro*, *Capsicum* annexin p35 inhibited porcine pancreatic phospholipase (PLA) A_2_ [72]. A tomato annexin was recently found to interact with a Universal Stress Protein in drought responses [73]. Finally, data from Huang *et al*. [68] eliminated several CDPKs, CIPKs and a wall-associated kinase as interacting partners for GbANN6. Such studies will be of significant value in elucidating the cellular functions of annexins.

## 7. Post-Translational Modifications

Function in signalling may also be regulated by post-translational modification. Conservation of two cysteine residues affords both S-nitrosylation and S-glutathionylation of AtANN1, with S-glutathionylation triggered by ABA [74,75]. This modification would impede Ca^2+^-mediated membrane association [75] and perhaps help terminate a Ca^2+^ signal. Participation of annexins in NO-regulated Ca^2+^ signalling now needs to be tested for.

Phosphorylation of plant annexins has been reported, with that of a *Brassica napus* annexin evident at the N terminal of the second annexin repeat [76]. Rice annexins interact with a MAPKK and Ste20-related protein kinase [77] while *Gossypium hirsutum* GhANN1 is phosphorylated by a plasma membrane-associated kinase [78]. AtANN1 can undergo phosphorylation by SnRK2s in ABA signalling [79] and, in common with annexins of other species, its transcript is upregulated by ABA [13,17,57,75]. Exactly how AtANN1’s phosphorylation fits into drought or salinity-induced signalling is unknown but it would be useful to re-examine the transport characteristics of recombinant AtANN1 with phosphorylation as a variable. Phosphorylation enhances AtANN1’s very weak *in vitro* peroxidase activity [55] but this activity could be due to contaminating proteins [16]. Exogenous H_2_O_2_ strongly suppresses *AtANN1* transcription in roots, most probably through the UPBEAT1 (AtUPB1) transcription factor [28,53]. However, H_2_O_2_ upregulates peroxidase activity [80] and so it appears that AtANN1’s main function in response to this ROS is as a component of Ca^2+^ transport rather than to de-toxify ROS [53].

## 8. Conclusions

With the capacity of annexins to modulate Ca^2+^ transport and cytosolic Ca^2+^ tested, the range of annexin involvement in calcium signalling now needs to be explored. The association of annexins with intracellular membranes means that research should not be limited just to cytosolic calcium, but needs to be extended to mitochondria, chloroplasts and the nucleus. There is a clear need to answer the longstanding question of whether annexins such as MtANN1 function in symbiotic nuclear calcium signalling [81,82,83,84]. Similarly, the role of annexins in immunity requires further attention. More tools are now available with which to test for annexin function in Ca^2+^ signalling and it is hoped that more plant researchers will take up this challenge.

## References

[1] McAinsh M.R., Pitman J.K. (2009). Shaping the calcium signature. New Phytol..

[2] Jammes F., Hu H.-C., Villiers F., Boueten R., Kwak J.M. (2011). Calcium-permeable channels in plants. FEBS J..

[3] Swarbreck S.M., Colaço R., Davies J.M. (2013). Update on plant calcium-permeable channels. Plant Physiol..

[4] Dadacz-Narloch B., Kimura S., Kurusu T., Farmer E.E., Becker D., Kuchitsu K., Hedrich R. (2013). On the cellular site of two-pore channel TPC1 action in the Poaceae. New Phytol..

[5] Chen Z.-H., Hills A., Bätz U., Amtmann A., Lew V.L., Blatt M.R. (2012). Systems dynamic modelling of the stomatal guard cell predicts emergent behaviors in transport, signalling, and volume control. Plant Physiol..

[6] Michard E., Lima P.T., Borges F., Silva A.C., Portes M.T., Carvalho J.E., Gilliham M., Liu L.H., Obermeyer G., Feijó J. (2011). Glutamate receptor-like genes form Ca^2+^ channels in pollen tubes and are regulated by pistil D-serine. Science.

[7] Vincill E.D., Bieck A.M., Spalding E.P. (2012). Ca^2+^ conduction by an amino acid-gated ion channel related to glutamate receptors. Plant Physiol..

[8] Tunc-Ozmedir M., Rato C., Brown E., Rogers S., Mooneyham A., Frietsch S., Myers C.T., Poulsen L.R., Malhó R., Harper J.F. (2013). Cyclic nucleotide gated channels 7 and 8 are essential for male reproductive fertility. PLoS One.

[9] Ma Y., Zhao Y., Walker R.K., Berkowitz G.A. (2012). Molecular steps in the immune signaling pathway evoked by plant elicitor peptides: Ca^2+^-dependent protein kinases, nitric oxide, and reactive oxygen species are downstream from the early Ca^2+^ signal. Plant Physiol..

[10] Ma Y., Walker R.K., Zhao Y., Berkowitz G.A. (2013). Linking ligand perception by PEPR pattern recognition receptors to cytosolic Ca^2+^ elevation and downstream immune signaling in plants. Proc. Natl. Acad. Sci. USA.

[11] Laohavisit A., Shang Z., Rubio L., Cuin T.A., Very A.-A., Wang A.H., Mortimer J.C., Macpherson N., Coxon K.M., Battey N.H. (2012). *Arabidopsis* annexin1 mediates the radical-activated plasma membrane Ca^2+^- and K^+^-permeable conductance in root cells. Plant Cell.

[12] Laohavisit A., Richards S.L., Shabala L., Chen C., Colaço R., Swarbreck S.M., Shaw E., Dark A., Shabala S., Shang Z.-L. (2013). Salinity-induced calcium signaling and root adaptation in *Arabidopsis* require the regulatory protein annexin1. Plant Physiol..

[13] Laohavisit A., Davies J.M. (2011). Annexins. New Phytol..

[14] Guelette B.S., Benning U.F., Hoffmann-Benning S. (2012). Identification of lipids and lipid-binding proteins in phloem exudates from *Arabidopsis thaliana*. J. Exp. Bot..

[15] Li B., Li D.-D., Zhang J., Xia H., Wang X.-L., Li Y., Li X.-B. (2013). Cotton AnnGh3 encoding an annexin protein is preferentially expressed in fibers and promotes initiation and elongation of leaf trichomes in transgenic *Arabidopsis*. J. Integr. Plant Biol..

[16] Clark G.B., Morgan R.O., Fernandez M.-P., Roux S.J. (2012). Evolutionary adaptation of plant annexins has diversified their molecular structures, interactions and functional roles. New Phytol..

[17] Feng Y.M., Wei X.K., Liao W.X., Huang L.H., Zhang H., Liang S.C., Peng H. (2013). Molecular analysis of the annexin gene family in soybean. Biol. Plant..

[18] Jami S.K., Clark G.B., Ayele B.T., Ashe P., Kirti P.B. (2012). Genome-wide comparative analysis of annexin superfamily in plants. PLoS One.

[19] Liu T.-W., Fu B., Niu L., Chen J., Wang W.-H., He J.-X., Pei Z.-M., Zheng H.-L. (2011). Comparative proteomic analysis of proteins in response to simulated acid rain in *Arabidopsis*. J. Proteome Res..

[20] Chu P., Chen H., Zhou Y.L., Li Y., Ding Y., Jiang L., Tsang E.W.T., Wu K., Huang S. (2012). Proteomic and functional analyses of *Nelumbo nucifera* annexins involved in seed thermotolerance and germination vigor. Planta.

[21] Fernandez M.B., Pagano M.R., Daleo G.R., Guevara M.G. (2012). Hydrophobic proteins secreted into the apoplast may contribute to resistance against *Phytophthora infestans* in potato. Plant Physiol. Biochem..

[22] Urbany C., Colby T., Stich B., Schmidt L., Schimdt J., Gebhardt C. (2012). Analysis of natural variation of the potato tuber proteome reveals novel candidate genes for tuber bruising. J. Proteome Res..

[23] Limpens E., Moling S., Hooiveld G., Pereira P.A., Bisseling T., Becker J.D., Küster H. (2013). Cell-and tissue-specific transcriptome analyses of *Medicago truncatula* root nodules. PLoS One.

[24] Yacoubi R., Job C., Belghazi M., Chaibi W., Job D. (2013). Proteomic analysis of the enhancement of seed vigour in osmoprimed alfalfa seeds germinated under salinity stress. Seed Sci. Res..

[25] Zhang Y., Xu L., Zhu X., Gong Y., Xiang F., Sun X., Liu L. (2013). Proteomic analysis of heat stress response in leaves of radish (*Raphanus sativus* L.). Plant Mol. Biol. Report..

[26] Zhou M.-L., Yang X.-B., Zhang Q., Zhou M., Zhao E.-Z., Tang Y.-X., Zhu X.-M., Shao J-R., Wu Y.-M. (2013). Induction of annexin by heavy metals and jasmonic acid in *Zea mays*. Funct. Integr. Genomics.

[27] Punwani J.A., Rabiger D.S., Drews G.N. (2007). MYB98 positively regulates a battery of synergid-expressed genes encoding filiform apparatus-localized proteins. Plant Cell.

[28] Tsukagoshi H., Busch W., Benfey P.N. (2010). Transcriptional regulation of ROS controls transition from proliferation to differentiation in the root. Cell.

[29] Laohavisit A., Davies J.M. (2009). Multifunctional annexins. Plant Sci..

[30] Obata T., Matthes A., Koszior S., Lehmann M., Araujo W.L., Bock R., Sweetlove L.J., Fernie A.R. (2011). Alteration of mitochondrial protein complexes in relation to metabolic regulation under short-term oxidative stress in *Arabidopsis* seedlings. Phytochemistry.

[31] Laohavisit A., Mortimer J.C., Demidchik V., Coxon K.M., Stancombe M.A., Macpherson N., Brownlee C., Hofmann A., Webb A.A.R., Miedema H. (2009). *Zea mays* annexins modulate cytosolic free Ca^2+^ and generate a Ca^2+^-permeable conductance. Plant Cell.

[32] Swisher J.F., Burton N., Bacot S.M., Vogel S.N., Feldman G.M. (2010). Annexin A2 tetramer activates human and murine macrophages through TLR4. Blood.

[33] Hofmann A., Proust J., Dorowski A., Schantz R., Huber R. (2000). Annexin 24 from *Capsicum annuum*. X-ray structure and biochemical characterization. J. Biol. Chem..

[34] Hu N.-J., Yusof A.M., Winter A., Osman A., Reeve A.K., Hofmann A. (2008). The crystal structure of calcium-bound annexin Gh1 from *Gossypium hirsutum* and its implications for membrane binding mechanisms of plant annexins. J. Biol. Chem..

[35] Breton G., Vazquez-Tello A., Danyluk J., Sarhan F. (2000). Two novel intrinsic annexins accumulate in wheat membranes in response to low temperature. Plant Cell Physiol..

[36] Gorecka K.M., Thouverey C., Buchet R., Pikula S. (2007). Potential role of annexin AtANN1 from *Arabidopsis thaliana* in pH-mediated cellular response to environment stimuli. Plant Cell Physiol..

[37] Santoni V., Rouquie D., Doumas P., Mansion M., Boutry M., Degand H., Dupree P., Packman L., Sherrier J., Prime T. (1998). Use of a proteome strategy for tagging proteins present at the plasma membrane. Plant J..

[38] Laohavisit A., Davies J.M. (2011). Annexins. Coding and Decoding of Calcium Signals in Plants.

[39] Laohavisit A., Brown A.T., Cicuta P., Davies J.M. (2010). Annexins: Components of the calcium and reactive oxygen signaling network. Plant Physiol..

[40] Kubista H., Hawkins T.E., Moss S.E. (1999). Annexin V mediates a peroxide-induced Ca^2+^-influx in B-cells. Curr. Biol..

[41] Balasubramanian K., Bevers E.M., Willems G.M., Schroit A.J. (2001). Binding of annexin V to membrane products of lipid peroxidation. Biochemistry.

[42] Kodavali P.K., Skowronek K., Koszela-Piotrowska I., Strzelecka-Kiliszek A., Pawlowski K., Pikula S. (2013). Structural and functional characterization of annexin 1 from *Medicago truncatula*. Plant Physiol. Biochem..

[43] Neumann E., Siemens P.M., Toensing K. (2000). Electroporative fast pore-flickering of the annexin V-lipid surface complex, a novel gating concept for ion transport. Biophys. Chem..

[44] Mortimer J.C., Coxon K.M., Laohavisit A., Davies J.M. (2009). Heme-independent soluble and membrane-associated peroxidase activity of a *Zea mays* annexin preparation. Plant Signal. Behav..

[45] Ito J., Heazlewood J.L., Millar A.H. (2006). Analysis of the soluble ATP-binding proteome of plant mitochondria identifies new proteins and nucleotide triphosphate interactions within the matrix. J. Proteome Res..

[46] Shang Z., Laohavisit A., Davies J.M. (2009). Extracellular ATP activates an *Arabidopsis* plasma membrane Ca^2+^-permeable conductance. Plant Signal. Behav..

[47] Demidchik V., Nichols C., Dark A., Oliynyk M., Glover B.J., Davies J.M. (2003). Is ATP a signaling agent in plants?. Plant Physiol..

[48] Demidchik V., Shang L., Shin R., Thompson E., Rubio L., Laohavisit A., Mortimer J.C., Chivasa S., Slabas A.R., Glover B.J. (2009). Plant extracellular ATP signalling by plasma membrane NADPH oxidase and Ca^2+^ channels. Plant J..

[49] Clark G., Roux S.J. (2011). Apyrases, extracellular ATP and the regulation of growth. Curr. Opin. Plant Biol..

[50] Carroll A.D., Moyen C., van Kesteren P., Tooke F., Battey N.H., Brownlee C. (1998). Ca^2+^, annexins, and GTP modulate exocytosis from maize root cap protoplasts. Plant Cell.

[51] Demidchik V., Shabala S.N., Coutts K.B., Tester M.A., Davies J.M. (2003). Free oxygen radicals regulate plasma membrane Ca^2+^ & K^+^-permeable channels in plant root cells. J. Cell Sci..

[52] Foreman J., Demidchik V., Bothwell J.H.F., Mylona P., Miedema H., Torres M.A., Linstead P., Costa S., Brownlee C., Jones J.D.G. (2003). Reactive oxygen species produced by NADPH oxidase regulate plant cell growth. Nature.

[53] Richards S.L., Laohavisit A., Mortimer J.C., Shabala L., Swarbreck S.M., Shabala S., Davies J.M. (2013). Annexin 1 regulates the H_2_O_2_-induced calcium signature in *Arabidopsis thaliana* roots. Plant J..

[54] Rentel M.C., Knight M.R. (2004). Oxidative stress-induced calcium signaling in *Arabidopsis*. Plant Physiol..

[55] Gorecka K.M., Konopka-Postupolska D., Hennig J., Buchet R., Pikula S. (2005). Peroxidase activity of annexin 1 from *Arabidopsis thaliana*. Biochem. Biophys. Res. Commun..

[56] Renew S., Heyno E., Schopfer P., Liszkay A. (2005). Sensitive detection and localization of hydroxyl radical production in cucumber roots and Arabidopsis seedlings by spin trapping electron paramagnetic resonance spectroscopy. Plant J..

[57] Lee S., Lee E.J., Yang E.J., Lee J.E., Park A.R., Song W.H., Park O.K. (2004). Proteomic identification of annexins, calcium-dependent membrane binding protein that mediate osmotic stress and abscisic acid signal transduction in *Arabidopsis*. Plant Cell.

[58] Chung J.S., Zhu J.K., Bressan R.A., Hasegawa P.M., Shi H.Z. (2008). Reactive oxygen species mediate Na^+^-induced SOS1 mRNA stability in *Arabidopsis*. Plant J..

[59] Thonat C., Mathieu C., Crevecoeur M., Penel C., Gaspar T., Boyer N. (1997). Effects of a mechanical stimulation of localization of annexin-like proteins in *Bryonia dioica* internodes. Plant Physiol..

[60] Clark G.B., Rafati D.S., Bolton R.J., Dauwalder M., Roux S.J. (2000). Redistribution of annexin in gravistimulated pea plumules. Plant Physiol. Biochem..

[61] Demir F., Horntrich C., Blachutzik J.O., Scherzer S., Reinders Y., Kierszniowska S., Schulze W.X., Harms G.S., Hedrich R., Geiger D. (2013). *Arabidopsis* nano-domain-delimited ABA signalling pathway regulates the anion channel SLAH3. Proc. Natl. Acad. Sci. USA.

[62] Chasserot-Golaz S., Vitale N., Umbrecht-Jenck E., Knight D., Gerke V., Bader M.F. (2005). Annexin 2 promotes the formation of lipid microdomains required for calcium-regulated exocytosis of dense-core vesicles. Mol. Biol. Cell.

[63] Lefebvre B., Furt F., Hartmann M.-A., Michaelson L.V., Carde J.-P., Sargueil-Boiron F., Rossignol M., Napier J.A., Cullimore J., Bessoule J.-J. (2007). Characterization of lipid rafts from *Medicago truncatula* root plasma membranes: A proteomic study reveals the presence of a raft-associated redox system. Plant Physiol..

[64] Liu P., Li R.-L., Zhang L., Wang Q.-L., Niehaus K., Baluska F., Samaj J., Lin J.-X. (2009). Lipid microdomain polarization is required for NADPH oxidase-dependent ROS signaling in *Picea meyeri* pollen tube tip growth. Plant J..

[65] Ning S.-B., Song Y.-C., van Damme P. (2002). Characterization of the early stages of programmed cell death in maize root cells by using comet assay and the combination of cell electrophoresis with annexin binding. Electrophoresis.

[66] Reina-Pinto J.J., Voisin D., Kurdyukov S., Faust A., Haslam R.P., Michaelson L.V., Efremova N., Franke B., Schreiber L., Napier J.A. (2009). Misexpression of FATTY ACID ELONGATION1 in the *Arabidopsis* epidermis induces cell death and suggests a critical role for phospholipase A2 in this process. Plant Cell.

[67] Huh S.M., Noh E.Y., Kim H.G., Jeon B.W., Bae K., Hu H.C., Kwak J.M., Park O.K. (2010). *Arabidopsis* annexins AnnAt1 and AnnAt4 interact with each other and regulate drought and salt stress responses. Plant Cell Physiol..

[68] Huang Y., Wang J., Zhang L., Zuo K. (2013). A cotton annexin protein AnxGb6 regulates fiber elongation through its interaction with actin 1. PLoS One.

[69] Wang Y.F., Fan L.M., Zhang W.Z., Zhang W., Wu W.H. (2004). Ca^2+^-permeable channels in the plasma membrane of *Arabidopsis* pollen are regulated by actin microfilaments. Plant Physiol..

[70] Wu J.-Y., Jin C., Qu H.-Y., Tao S.-T., Xu G.-H., Wu J., Wu H.-Q., Zhang S.-L. (2012). Low temperature inhibits pollen viability by alteration of actin cytoskeleton and regulation of pollen plasma membrane ion channels in *Pyrus pyrifolia*. Environ. Exp. Bot..

[71] Morgan R.O., Martin-Almedina S., Garcia M., Jhoncon-Kooyip J., Fernandez M.P. (2006). Deciphering function and mechanism of calcium-binding proteins from their evolutionary imprints. Biochim. Biophys. Acta.

[72] Hoshino T., Mizutani A., Chida M., Hidaka H., Mizutani J. (1995). Plant annexins form homodimer during Ca^2+^-dependent liposome aggregation. Biochem. Mol. Biol. Int..

[73] Loukehaich R., Wang T., Ouyang B., Ziaf K., Li H., Zhang J., Lu Y., Ye Z. (2012). SpUSP, an annexin-interacting universal stress protein, enhances drought tolerance in tomato. J. Exp. Bot..

[74] Lindermayr C., Saalbach G., Durner J. (2005). Proteomic identification of S-nitrosylated proteins in *Arabidopsis*. Plant Physiol..

[75] Konopka-Postupolska D., Clark G., Goch G., Debski J., Floras K., Cantero A., Fiolek B., Roux S., Hennig J. (2009). The role of annexin 1 in drought stress in *Arabidopsis*. Plant Physiol..

[76] Agrawa G.K., Thelen J.J. (2006). Large scale identification and quantitative profiling of phosphoproteins expressed during seed filling in oilseed rape. Mol. Cell. Proteomics.

[77] Rohila J.S., Chen M., Chen S., Chen J., Cerny R., Dardick C., Canlas P., Xu X., Gribskov M., Kanrar S. (2006). Protein-protein interactions of tandem affinity purification-tagged protein kinases in rice. Plant J..

[78] Andrawis A., Solomon M., Delmer D.P. (1993). Cotton fibre annexins: A potential role in the regulation of callose synthase. Plant J..

[79] Wang P., Xue L., Batelli G., Lee S., Hou Y.-J., van Oosten M.J., Zhang H., Tao W.A., Zhu J.-K. (2013). Quantitative phosphoproteomics identifies SnRK2 protein kinase substrates and reveals the effectors of abscisic acid action. Proc. Natl. Acad. Sci. USA.

[80] Yang Y.L., Zhang Y.Y., Lu J., Zhang H., Liu Y., Shi R.X. (2012). Exogenous H_2_O_2_ increased catalase and peroxidase activities and proline content in *Nitraria tangutorum* callus. Biol. Plant..

[81] Kovács I., Ayaydin F., Oberschall A., Ipacs I., Bottka S., Pongor S., Dudits D., Toth E.C. (1998). Immunolocalization of a novel annexin-like protein encoded by a stress and abscisic acid responsive gene in alfalfa. Plant J..

[82] De Carvalho-Niebel F., Lescure N., Cullimore J.V., Gamas P. (1998). The *Medicago truncatula MtAnn1* gene encoding an annexin is induced by nod factors and during the symbiotic interaction with *Rhizobium meliloti*. Mol. Plant Microbe Interact..

[83] De Carvalho-Niebel F., Timmers A.C.J., Chabaud M., Defaux-Petras A., Barker D.G. (2002). The Nod factor-elicited annexin *MtAnn1* is preferentially localised at the nuclear periphery in symbiotically activated root tissues of *Medicago truncatula*. Plant J..

[84] Talukdar T., Gorecka K.M., de Carvalho-Niebel F., Downie J.A., Cullimore J., Pikula S. (2009). Annexins—Calcium- and membrane-binding proteins in the plant kingdom. Potential role in nodulation and mycorrhization in *Medicago truncatula*. Acta Biochim. Pol..

